# Genetic Variants of *ANGPT1*, *CD39*, *FGF2* and *MMP9* Linked to Clinical Outcome of Bevacizumab Plus Chemotherapy for Metastatic Colorectal Cancer

**DOI:** 10.3390/ijms22031381

**Published:** 2021-01-30

**Authors:** María Gaibar, Miguel Galán, Alicia Romero-Lorca, Beatriz Antón, Diego Malón, Amalia Moreno, Ana Fernández-Santander, Apolonia Novillo

**Affiliations:** 1 Department of Health Sciences, Health Sciences Faculty, European University of Madrid, Villaviciosa de Odón, 28670 Madrid, Spain; maria.gaibar@universidadeuropea.es (M.G.); miguel-galan-burgos@hotmail.com (M.G.); 2 Department of Medicine, Health Sciences Faculty, Universidad Europea de Madrid, Villaviciosa de Odón, 28670 Madrid, Spain; alicia.romero@universidadeuropea.es (A.R.-L.); ana.fernandez@universidadeuropea.es (A.F.-S.); 3 Department of Oncology, University Hospital of Fuenlabrada, Fuenlabrada, 28942 Madrid, Spain; beatriz.anton@salud.madrid.org (B.A.); diego.malon@salud.madrid.org (D.M.); 4 Department of Pathological Anatomy, University Hospital of Fuenlabrada, Fuenlabrada, 28942 Madrid, Spain; amorenot@salud.madrid.org; 5 Department of Pre-Clinical Dentistry, Health Sciences Faculty, Universidad Europea de Madrid, Villaviciosa de Odón, 28670 Madrid, Spain

**Keywords:** bevacizumab, colorectal cancer, angiogenesis, polymorphisms

## Abstract

Angiogenesis pathway genes show substantial genetic variability causing inter-individual differences in responses to anti-angiogenic drugs. We examined 20 single nucleotide polymorphisms (SNPs) in 13 of these genes to predict tumour response and clinical outcome measured as progression free survival (PFS) and overall survival (OS) in 57 patients with metastatic colorectal cancer (mCRC) given bevacizumab plus chemotherapy. SNPs were detected (iPLEX® Assay) in genomic DNA extracted from formalin-fixed paraffin-embedded tumour specimens. The variant allele *CD39* rs11188513 was associated with a good tumour response (*p* = 0.024). Patients homozygous for the wild-type allele *FGF2* rs1960669 showed a median PFS of 10.95 months versus 5.44 months for those with at least one variant allele-A (HR 3.30; 95% CI: 1.52–7.14; *p* = 0.001). Patients homozygous for wild-type *MMP9* rs2236416 and rs2274755 showed a median PFS of 9.48 months versus 6 and 6.62 months, respectively, for those with at least one variant allele (*p* = 0.022, *p* = 0.043, respectively). OS was also lengthened to 30.92 months (*p* = 0.034) in carriers of wild-type *ANGPT1* rs2445365 versus 22.07 months for those carrying at least one variant allele-A. These gene variants were able to predict clinical outcome and tumour response in mCRC patients given bevacizumab-based therapy.

## 1. Introduction

Colorectal cancer (CRC) is the fourth most common cancer and the fifth leading cause of cancer death worldwide, with an estimated 1.85 million new cases and 861,663 deaths recorded in 2018 [1]. Metastasis is the most lethal characteristic of CRC and accounts for 90% of all deaths registered in colon cancer patients [2]. Metastatic CRC (mCRC) is a complex disease that is largely influenced by lifestyle and dietary factors, and is also linked to socioeconomic status. Thus, the management of this disease requires new-age practices in a predictive, preventive, and personalized medicine (PPPM) approach [1]. Recent studies have suggested that inter-individual genetic variations may significantly affect an individual’s predisposition to and risk of developing mCRC [3]. Further, there is also increasing evidence that single nucleotide polymorphisms (SNPs) may be used as biomarkers of the genetic background of mCRC patients to predict their individual therapeutic response and prognosis [4,5].

Bevacizumab (BVZ) is a humanized monoclonal antibody that targets vascular endothelial growth factor (VEGF). This agent is used in combination with chemotherapy (CT) to treat mCRC [6]. BVZ works by inhibiting the actions of VEGF, causing regression of existing tumour vasculature and preventing the development of new blood vessels, thereby inhibiting tumour growth. At the molecular level, VEGF and its receptor (VEGFR) play major roles in the angiogenesis that characterizes advanced cancer. Several authors have reported significant improvements in progression-free survival (PFS), overall survival (OS) and response rate (RR) in mCRC patients treated with CT + BVZ compared to CT alone [7,8,9,10,11,12]. However, not all patients respond in the same way to treatment probably through the development of treatment resistance mechanisms involving activation of proangiogenic pathways or tumour recruitment of cells that produce proangiogenic factors, recruitment of hematopoietic cells and inflammatory cells into the tumour, and heterogeneity of genetically unstable tumour cells [13,14].

Several clinical studies in patients with mCRC have shown a relationship between variation in the SNPs of genes involved directly or indirectly in the angiogenesis pathway and the response to BVZ. Variants of these genes have also been associated with the aetiology and clinical outcomes of mCRC [15,16,17,18,19,20]. However, while basic studies have revealed an essential role of VEGF-dependent and independent angiogenesis pathway genes in the aetiology and clinical outcome of mCRC, there is still scarce knowledge regarding differences the efficacy of BVZ treatment and resistance to this treatment observed among patients [21]. In the present study, we hypothesized that variations in genes directly or indirectly regulating angiogenesis processes, such as those coding for growth factors, cytokine signalling molecules, angiopoietin and components of the adenosine pathway, could help predict the efficacy of anti-VEGF drug-containing chemotherapy in patients with mCRC. To this end, we examined whether several single-nucleotide polymorphisms (SNPs) in the major VEGF-dependent and independent angiogenic pathway genes *A2BR*, *ANGPT1*, *ANGPT-2*, *CCL5*, *CD39*, *EDN1*, *FGF2*, *IGF1*, *MKNK1*, *MMP9*, *NT5E*, *TOP1* and *VEGF-A* could correlate with treatment efficacy and clinical outcomes in mCRC patients receiving BVZ in combination with CT. Clinical outcomes were measured in terms of tumour response, PFS, OS and RR.

## 2. Results

The study cohort was comprised of 57 patients with mCRC. The primary tumour site was the proximal colon (left-sided) in 70% of patients (40/57) and the distal colon (right-sided) in 30% (17/57). In 55 of the patients, tumours were in advanced stages (96% stage III and IV) and with *KRAS* mutated (76%, 42/55) at diagnosis. The mean number of sites showing metastases was 2 (range 1 to 5). Mean age at the time of diagnosis was 61.8 years (range 27 to 81). Most patients were male (41/57; 72%) (Table 1).

Only 35% of the patients underwent surgical resection as the primary treatment option. All patients were given CT + BVZ. In more than half of the patients this regimen was the first-line option (58.9%, 33/57); the most frequently used first line associated CT was FOLFOX (47%, 27/57) (Table 1). A high percentage (79%) of patients did not require BVZ dose reduction. In only 11% of patients did treatment have to be withdrawn as deemed necessary by the responsible physician because of a lack of response or high toxicity of the drug. As secondary effects of BVZ treatment, only 13% (7/57) of patients experienced high toxicity (type III), while in 87% of patients, toxicity was classified as type I or II (50/57). Main side effects recorded were hypertension in 34% (19/57) of patients, intestinal perforation in 11% (6/57) and bleeding (18/57) in 32%, mainly epistaxis followed by rectal fistulas and haemorrhoids.

Median follow-up duration was 28.5 months. 61% of the patients were dead at the end of this study; median PFS and OS were 11.09 months and 34.79 months, respectively. Thirty patients (52.6%) showed a response to the treatment while tumour progression was recorded in 27 patients (47.4%) (Table 1).

### 2.1. Gene Variants

Genotyping quality control by direct DNA-sequencing provided a genotype agreement of 99%. Genotyping was successful in 97.98% of cases for each polymorphism examined, except for *MMPA*-rs17577 and *CDKAL1*- rs7453577, which were excluded from further analysis. Genotype frequencies of the 20 SNPs of the 13 genes investigated in this project are summarized in Table 2. Highest wild-type homozygosity rates were observed for *ANGPT-2* rs10102851 (92.98%), *VEGF-A* rs3025039 (75.44%), *FGF2* rs1960669 (82.14%), *MMP9* rs2236416 (75.44%), *MMP9* rs2274755 (76.78%), *EDN1* rs5370 (60.71%), *CCL5* rs2280789 (82.46%), *TOP1* rs34282819 (77.19%), *ANGPT1* rs2445365 (57.89%) and *MKNK1* rs8602 (53.70%) (Table 2). For other genes, most patients were carriers of the variant allele in homozygosis or heterozygosis: *CD39* rs11188513 (89.47%), *NT5E* rs2229523 (94.64%), *ANGPT2* rs1375668 (89.29%), *ANGPT2* rs2515462 (92.98%), *VEGFA* rs833061 (82.46%) and *IGF1* rs6220 (92.86%). Further, around 50% of patients were heterozygous for the *VEGFA* polymorphisms rs833061, rs833068 and rs833069, and for *TOP1* rs6072249 and *A2BR* rs2015353. At a glance classification of the genes analysed in this study according to their Gene Ontology-Biological processes are provided in Table 2. This classification revealed that all the SNPs analysed affect genes directly or indirectly related to angiogenesis, which is the main biological process blocked by BVZ.

All the allele frequencies of the genes examined showed good agreement with Hardy-Weinberg equilibrium. In the univariate analysis of SNP frequencies observed in the patients, only for the variant allele of *CD39* rs11188513 were significant differences detected in the presence of the VV (TT) genotype (OR 3; 95% CI: 1.015–8.864; *p* = 0.047), versus WT homozygous or heterozygous (CT/CC) patients. None of the other tested polymorphisms or other characteristics analysed (Table 1) were significantly different in our cohort of patients. The genetic heterogeneity of tumours was addressed calculating the number of mutated genes according to tumour stage (Table 3). Patients in III and IV stage accumulated the highest number of genes with mutations. For more information about names of mutated genes and ID of SNPs investigated, see supplementary Table S1. Of interest, the variant *ANGPT1* rs2445365 was present in 19 of 39 patients in stage IV.

### 2.2. Gene Variants and Tumour Response 

To estimate the effects of the SNPs on the therapeutic efficacy of CT+BVZ, we determined whether there was a tumour response or not to 6 months of treatment according to the genotypes of the different polymorphisms. Thus, we compared the frequencies of each polymorphism in the two groups of patients responders (R) and non-responders (nR) by performing a univariate analysis between patients carrying: a) at least one variant allele (VV or W/V), and patients carrying two wild-type alleles (WW); and b) patients carrying two variant alleles (VV) and those carrying none or a single variant allele (W/V or WW). In this analysis, the variant *CD39* rs11188513 was significantly associated with a good tumour response to treatment. Thus, patients with the *CD39* rs11188513 variant genotype were more likely to belong to the group of patients responding to CT+BVZ treatment (62.07%) than the group of nR patients, who were heterozygous or homozygous for the wild-type (C) allele (67.86%) (*p* = 0.024). The frequencies of the different genotypes of the other polymorphisms did not differ significantly between responders and non-responders (data not shown).

### 2.3. Gene Variants, Progression-Free Survival and Overall Survival

**Figure 1:**
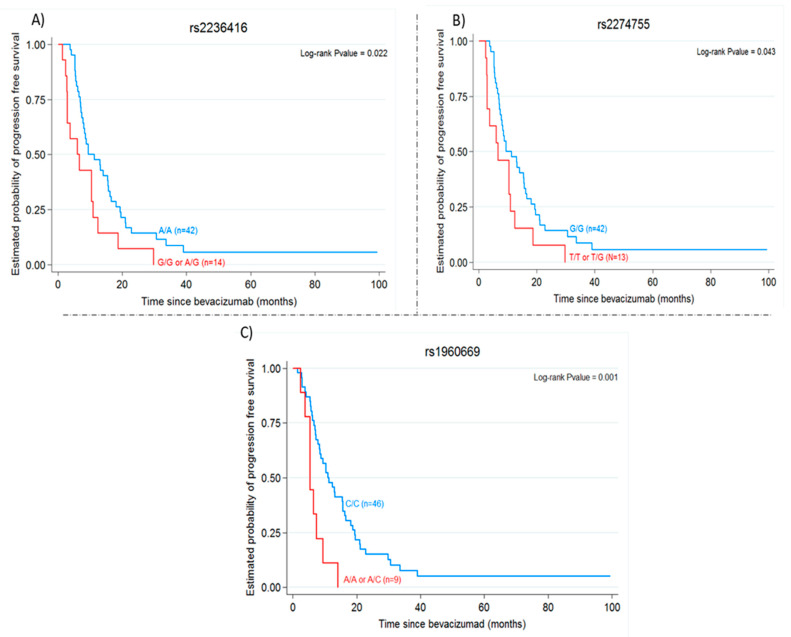
(**A**) PFS in response to bevacizumab treatment in patients with *MMP9* rs2236416 A/A vs. A/G /GG (9.48 vs. 6 months, *p* = 0.022). (**B**) PFS in response to bevacizumab treatment in patients with *MMP9* rs2274755 G/G vs. G/T /TT (9.48 vs. 6.82 months, *p* = 0.043). (**C**) PFS in response to bevacizumab treatment in patients with *FGF2* rs1960669 C/C vs. C/A /AA (10.95 vs. 5.44 months, *p* = 0.001).

In the univariate analysis, the wild-type allele of *FGF2* rs1960669, and wild-type alleles of *MMP9* rs2236416 and *MMP9* rs2274755 were found to be significantly associated with PFS. In the case of *FGF2* rs1960669, CC homozygous patients showed a median PFS of 10.95 months compared to 5.44 months for patients carrying at least one variant allele-A (HR 3.30; 95% CI: 1.52–7.14; *p* = 0.001). For the *MMP9* SNPs rs2236416 and rs2274755, patients homozygous for the wild-type allele showed a median PFS of 9.48 months versus 6 and 6.62 months, respectively, for patients carrying at least one variant allele (*p* = 0.022, *p* = 0.043, respectively) (see Table 4, Figure 1). No other significant correlations with PFS were observed for the other SNPs.

**Figure 2:**
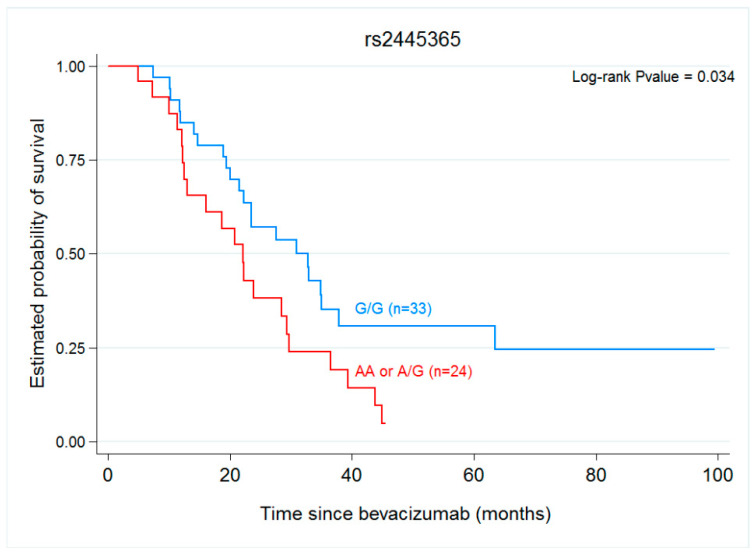
OS in response to bevacizumab treatment in patients with *ANGPT1* rs2445365 G/G vs. G/A/AA (30.92 vs. 22.07 months, *p* = 0.034).

Overall survival was found to be significantly (*p* = 0.034) longer in carriers of the wild-type *ANGPT1* rs2445365 allele (30.92 months) than in those carrying at least one variant allele-A (22.07 months) (Table 4 and Figure 2). None of the other variants were associated with OS (Table 4, Figure 2).

## 3. Discussion

In combination with chemotherapy, the widely used monoclonal antibody against VEGF, bevacizumab, shows differences in efficacy among cancer types and also among patients with the same cancer type [6,23,24,25]. Hence, following CT+BVZ, several studies have shown increases in the response rate, progression-free survival and overall survival in patients with mCRC compared to patients not treated with BVZ [7,8,9,11,12] and a significantly improved pCR (pathological complete response) in breast cancer patients [24]. Further, the findings of clinical studies indicate that the efficacy of BVZ as an antiangiogenic agent and its acquired resistance vary widely among mCRC patients [21]. This has prompted a search for biomarkers to validate the efficacy of this treatment and predict treatment outcomes.

In the past few decades, it has been suggested that some polymorphisms in VEGF-dependent and non-VEGF-dependent genes could be responsible for better clinical outcomes in patients with mCRC receiving CT+BVZ as first line treatment [24,26]. Further, studies in different cancer types (e.g., breast cancer) have also suggested that genetic variants affecting regulatory regions besides coding regions, could be of special interest in this context [24]. As angiogenic mechanisms are thought to play a role in resistance to BVZ, we explored a panel of SNPs with implications for tumour angiogenic pathways that could help predict an individual’s response to treatment. We selected 20 SNPs of 13 genes based on genetic variants described to affect a patient’s response to BVZ treatment in different type of cancers. These were SNPs located in regulatory regions able to modify gene expression (intron, 3´UTR, 5´UTR) and non-synonymous SNPs in coding regions (that modify the coded aminoacids). Overall allele frequencies of the 20 SNPs examined in our mCRC patients were comparable to frequencies reported for healthy European populations [22].

An interesting finding of our study was the relationship detected between the SNP *CD39* rs11188513 and the tumour response to treatment. This gene plays a role in the adenosine pathway and it is well established that extracellular adenosine has a potent immunosuppressive and angiogenic effect in the tumour microenvironment. Extracellular adenosine is autonomously produced via CD39 and CD73, expressed mainly on the surface membrane of cancer cells, B cells or regulatory T cells (Tregs). This adenosine not only stimulates cancer cells through its receptor A2BR but also regulates tumour-infiltrating immune cells through A2AR and A2BR [27,28,29]. In our study, patients homozygous for the variant allele *CD39* rs11188513 showed a good respond to treatment measured as non-tumour progression after 6 months of BVZ treatment. However, this response failed to impact PFS and OS. The effect of the *CD39* rs11188513 variant on the outcome of BVZ treatment has been explored by other authors with results in agreement with those detected here. Thus, Tokunaga et al. showed that patients with any wild-type rs11188513 allele (C) showed a worse response to BVZ treatment in terms of PFS and OS compared to patients with the VV genotype [30]. In this study, 451 mCRC patients were divided into 3 groups: control group (treated with FOLFIRI + cetuximab), analysis group (treated with FOLFIRI + BVZ) and validation group (largest group of patients treated with FOLFIRI + BVZ). It emerged that *CD39* rs11188513 was a strong predictor of BVZ treatment outcome in patients treated with FOLFIRI + BVZ. The *CD39* rs11188513 SNP is located in the 3’-UTR region of the gene, which is considered a binding site for miR-155 that could regulate the function of CD39 [31,32]. Tokonuga et al. also proposed it could work as a tag SNP and thus affect functional effects through related polymorphisms at other loci of *CD39* [30]. Although the effect of this SNP of *CD39* on phenotypic change remains unknown, several authors have suggested CD39 could be a checkpoint inhibitor target in that it prevents adenosine’s immunosuppressive effects, thus playing an important role in tumour progression [33]. Recently, a new role was identified for this SNP as a prognostic and predictive biomarker of BVZ treatment outcome [30]. Further work focusing on this SNP is warranted.

The explanation of inter-individual differences in the efficacy of BVZ as an anti-angiogenic drug to treat mCRC has not been well established yet. In our study, the *MMP9* polymorphisms rs2236416 and rs2274755 were related to PFS following CT+BVZ treatment. MMP9 mainly acts as a collagenase degrading type IV collagen, and is critical for tumour cell growth, migration, invasion, metastasis and angiogenesis [34,35]. Based on its broad functions, several studies and meta-analyses have identified MMP9 as a potential biomarker in various cancers [36,37,38]. Further, genetic variation influencing MMP9 expression could contribute to cancer susceptibility. Numerous *MMP9* polymorphisms have been reported, of which several are considered functional. However, the roles of many of these SNPs remain unclear. In terms of genetic variability and clinical outcome of bevacizumab treatment, data for SNPs of genes encoding MMPs are scarce. In the present study, based on data from Makhoul et al. we explored the impact of the *MMP9* polymorphisms rs2236416 and rs2274755 on the response to CT+BVZ shown by patients with mCRC. These authors showed that these two SNPs of the *MMP9* gene and its protein could influence tumour susceptibility to BVZ [24]. In effect, patients with these genetic variants were found more likely to show a pathologic complete response (pCR) in a prospective phase II trial testing the use of BVZ as neoadjuvant to chemotherapy in breast cancer patients. Here, tumours homozygous for the wild-type alleles of *MMP9* rs2236416 and rs2274755 showed a median PFS of 9.48 months, and carrying at least one variant allele led to a reduced median PFS of 6 and 6.62 months, respectively (*p* = 0.022, *p* = 0.043, respectively). The *MMP9* rs2274755 locus is located in an intron (boundary) of the MMP9 gene and occurs at the third base of the fourth intron. It therefore shows the potential to influence RNA splicing. The functional relevance of this SNP needs to be further investigated. While this *MMP9* rs2274755 has been investigated in several studies, none of these studies have examined its impact on the efficacy of BVZ treatment in mCRC patients, and available data suggest that this SNP might be involved in the development of asthma [39]. *MMP9* rs2236416 is an intron variant, and a significantly lower frequency of its wild-type (A) allele has been reported among individuals with Henoch-Schönlein purpura nephritis (HSPN) [40]. No functional data are available for this polymorphism. Although the precise molecular mechanism underlying our observations is unclear, a possible explanation is that the two variants of the *MMP9* gene may lead to the modified expression of this gene in mCRC patients with a negative impact on their response to BVZ treatment.

A further finding of our study was a significant association between *FGF2* rs1960669, an intronic tag-SNP, and PFS detected in a univariate analysis. This finding is especially interesting given the functional role of fibroblast growth factor (FGF)-2 in tumour biology with important implications for cancer therapies and clinical outcomes. FGF2 is a potent angiogenic molecule involved in tumour progression, and is one of several growth factors with a central role in ovarian cancer [41]. FGF2 has been associated with the regulation of tumour angiogenesis and metastasis, and has been positively correlated with epidermal growth factor (EGF) and IGF in breast cancer [42]. Abnormally high concentrations of FGF2 have been found in the serum of patients with active metastatic cancers and these have been shown to correlate significantly with extent of disease, clinical status and mortality risk [43]. Makhoul et al. showed that *FGF2* rs1960669 was associated with a pathologic complete response in their prospective phase II study of the use of BVZ as neoadjuvant to chemotherapy in breast cancer patients [24]. In future work, it will be interesting to address the role of *FGF2* rs1960669 in mCRC cancer survival and prognosis. If the efficacy of BVZ can be predicted according to individual genotype this could help with novel approaches to risk stratification or the use of anti-angiogenic treatment strategies. We speculate that the *FGF2* variant investigated here could influence the expression of specific isoforms, which might then uniquely influence tumour progression in response to BVZ.

VEGF-independent angiogenic signalling plays an important role in the development of colorectal cancer. A molecule involved in this pathway is ANGPT1. Our results indicate that OS is improved in patients carrying the wild-type *ANGPT1* rs2445365 variant while none of the other SNPs examined had any impact on this clinical parameter. While this may appear a shortcoming of our study, it does support the fact that angiogenesis inhibitors targeting the VEGF signalling pathway, such as BVZ, show significant therapeutic efficacy in various cancers [23]. In cancer patients, the treatment response is frequently transient and this could be the consequence of resistance to angiogenesis inhibition, adaptive resistance or intrinsic non-responsiveness [14]. These modes of resistance could be attributed to the inherent heterogeneity of genetically unstable tumour cells, the presence of redundant angiogenic factors, and the recruitment of hematopoietic cells and inflammatory cells into the tumour mass [14]. Thus, genetic variations in *ANGPT* genes may lead to altered gene production and result in their activation/inactivation. SNPs of the *ANGPT1* gene have been associated with the risk of autoimmune diseases, juvenile idiopathic arthritis, and portopulmonary hypertension [44,45], and also with the risk and clinical outcome of colorectal cancer (rs1954727) [46]. Makhoul et al. showed that *ANGPT1* rs2445365 was linked to a pathologic complete response (pCR) in their prospective phase II study of the use of bevacizumab as neoadjuvant to chemotherapy in breast cancer patients [24]. In our study, we observed that the wild-type allele of *ANGPT1* rs2445365, might improve the overall survival of mCRC patients treated with CT + BVZ. This SNP is located in the intron region of the *ANGPT1* gene and while the impact of this SNP on phenotypic change is still unknown, this finding merit further investigation. There is evidence to suggest that the ANGPT-TIE (ANGPT-TEK receptor) pathway is crucial for angiogenesis and vascular homeostasis in a VEGF-independent manner, and also of a link between angiogenesis and inflammation pathways [47]. The up-regulation of ANGPT1 in many cancers suggests this gene is strongly associated with tumour malignancy [47], and this could mean that *ANGPT1* rs2445365 affects the activity of this protein and that activation of angiopoietin-1/Tek signalling in the vasculature could induce metastasis. Further, several studies have reported an inhibiting effect of ANGPT1 on pathologic vascular expansion, suggesting its function as a tumour suppressor in several cancers, including CRC [48,49,50,51]. These findings suggest the effects of ANGPT1 on genetic tumour characteristics and prognosis might be cancer-specific and also dependent on other angiogenesis-related genes.

This study has limitations, as for instance, its rather small sample size and the heterogeneous nature of tumour tissue and clinical treatment. The potential clinical implications of these SNPs raise the need to perform larger studies with more patients and homogeneous clinical treatments. In case of further studies would sustain the usefulness of these SNPs as biomarkers, the genotyping would be very useful to select patients with probably good prognosis to treatment and to avoid side effects in those with more risks to suffer them. This personalized medicine would be a good and cheap strategy with easy implementation in the daily clinical to improve the treatment of mCRC with antiangiogenic drugs as BVZ.

## 4. Materials and Methods 

### 4.1. Design and Patients

The study population comprised 57 patients with histologically confirmed mCRC who were treated at the Oncology Department of the University Fuenlabrada Hospital between 2009 and 2019. They received bevacizumab in combination with chemotherapy (FOLFOX or FOLFORIFI, Xeloda, 5-Fluoroacil, or Irinotecan). Patient data were collected retrospectively through chart review by a medical oncologist (Dr. D Malón). The tumour biopsy site (left-sided colon or right-sided colon) and the type of biopsy (surgical specimen (both excisional and incisional), core needle biopsy) were obtained from medical records. This study was conducted at the Department of Oncology, University Hospital of Fuenlabrada, Madrid, Spain, in accordance with the Declaration of Helsinki and the International Conference on Harmonisation (ICH) Good Clinical Practice. Approval was obtained from our Hospital’s Ethics Committee (identification code: APR 15/38, August 2015) and Research Committee of the European University (identification code: CIPI/18/106, April 2018). Written informed consent to participate in this study was obtained from all patients.

Baseline clinical examinations and staging CT-scans were performed each three weeks after starting treatment. Response Evaluation Criteria in Solid Tumors (RECIST) were used to determine the response rate. Patients’ responses were categorized as good (complete or partial response after 6 months of BVZ treatment, R group) or no response (stable or progressive disease after 6 months of BVZ treatment, nR group). Responsible physicians decided upon the maintenance of treatment. The study endpoints were progression-free survival (PFS), and overall survival (OS). PFS was calculated as the time from the first day of treatment until the first observation of disease progression or any-cause death. OS was defined as the time from the first day of treatment to any-cause death. If a patient remained alive and their cancer had not progressed, PFS and OS were censored at the time of last follow-up.

### 4.2. Candidate Polymorphisms

Genes and polymorphisms known to regulate VEGF-dependent and independent angiogenesis were selected based on published research and public databases. The predefined criteria used were: a) scientific information available to support the involvement of the gene in angiogenesis signalling pathways; b) polymorphisms published as biomarkers of a response to BVZ treatment in cancer, mainly CRC; and c) minor allele frequency > 5% in Caucasians. 20 SNPs of 13 genes were analysed: *A2BR* rs2015353, *ANGPT1* rs2445365, *ANGPT2* (rs10102851, rs1375668, rs2515462), *CCL5* rs2280789, *CD39* rs11188513, *EDN1* rs5370, *FGF2* rs1960669, *IGF1* rs6220, *MKNK1* rs8602, *MMP9* (rs2236416, rs2274755), *NT5E* rs2229523, *TOP1* (rs34282819, rs6072249), *VEGF-A* (rs3025039, rs833061, rs833068, rs833069). At a glance classification of the genes analysed in this study according to their Gene Ontology-Biological processes are provided in Table 2. This classification revealed that all the SNPs analysed affect genes directly or indirectly related to angiogenesis.

### 4.3. Tumour DNA Extraction and Genotyping

Surgical (i.e., either incisional or excisional biopsies that required a surgical procedure) and core needle biopsies were processed using standard techniques (fixation in 10% neutral buffered formalin and paraffin-embedding). Once a diagnosis was established in histologic and/or immunohistologic staining profiles, the residual materials in the formalin-fixed paraffin-embedded (FFPE) tissue blocks were used for molecular analysis. When multiple tissue blocks were available, the one with the highest tumour cellularity was chosen, without additional tumour microdissection or enrichment. 

For each paraffin-embedded block, 3–5 slices of 5 μm were collected using an ultramicrotome (Leica EM UC7, Wetzlar, Germany), always discarding the first slices. DNA extraction from these samples was performed using a Maxwell R 16 FFPE plus Lev DNA Purification Kit (Promega, Madison, WI, USA) following the manufacturer’s recommendations. The DNA samples were re-suspended in DNAse-free water (50 μL). The concentration and quality of DNA samples were quantified with the Nanodrop 2000 (Thermo Fisher Scientific, Wilmington, DE, USA), and only samples with a DNA concentration of 50 ng/μL or more that met standard quality criteria were selected for genotyping. The DNA quality criterion used was the ratio of the absorbance at 260 nm divided by the reading at 280 nm. Good-quality DNA has a A260/A280 ratio of 1.8–2.0. Good quality samples were stored at −20 °C in 96 well plates. 

Genotyping of 20 SNPs was conducted by the Spanish National Genotyping (CeGen-PRB2-ISCIII, www.cegen.org) using the iPlex® Gold chemistry and MassARRAY platform, according to manufacturer’s instructions (Agena Bioscience, San Diego, CA, USA). Genotyping assays were designed on GRCh38 version using the Agena Bioscience MassARRAY Assay Designer 4.0 software (Agena Bioscience, San Diego, CA, USA). The 20 SNPs were genotyped in 2 independent assays. PCR reactions were set up in a 5 µl volume and contained 20 ng of template DNA, 1× PCR buffer, 2 mM MgCl2, 500 µM dNTPs and 1 U/reaction of PCR enzyme. Reagents for PCR were for Agena Bioscience (San Diego, CA, USA). A pool of PCR primers was made at a final concentration of each primer of 100 nM. The thermal cycling conditions for the reaction consisted of an initial denaturation step at 94 °C for 2 min, followed by 45 cycles of 94 °C for 30 s, 56 °C for 30 s and 72 °C for 1 min, followed by a final extension step of 72 °C for 1 min. Unincorporated dTNPs were dephosphorylated using shrimp alkaline phosphatase (SAP), so PCR products were treated with 0.6 U shrimp alkaline phosphatase by incubation at 37 °C for 40 min, followed by enzyme inactivation by heating at 85 °C for 5 min.

The iPLEX Gold reactions were set up in a final 9 µl volume and contained 0.222x iPLEX buffer Plus, 0.5x iPLEX termination mix and 0.5x iPLEX enzyme. An extension primer mix was made to give a final concentration of each primer between 0.73 µM and 1.46 µM. The thermal cycling conditions for the reaction included an initial denaturation step at 94 °C for 30 s, followed by 40 cycles of 94 °C for 5 s, with an internal 5 cycles loop at 52 °C for 5 s and 80 °C for 5 s, followed by a final extension step of 72 °C for 3 min. The next step is to desalt the iPLEX Gold reaction products with Clean Resin (Agena Bioscience, San Diego, CA, USA) following the manufacturer’s protocol. The desalted products were dispensed onto a 384 Spectrochip II using an RS1000 Nanodispenser and spectra were acquired using the MA4 (Agena Bioscience, San Diego, CA, USA) mass spectrometer, followed by manual inspection of spectra by trained personnel using MassARRAY Typer software v4.0.26 (Agena Bioscience, San Diego, CA, USA). All assays were performed in 384-well plates, including negative controls and a trio of Coriell samples (Na10861, Na11994 and Na11995) for quality control. Two samples and two SNPs (*MMPA*-rs17577 and *CDKAL1*-rs7453577) were discarded because of low reproducibility, and not considered for further analysis. The SNP rs444903, that represent an intergenic region, was not considered for further analysis. Internal controls showed 100% reproducibility and genotyping success.

Allele and genotype frequencies for all the SNPs (13 genes and 20 SNPs, see Table 2) were estimated by direct counting; genotype and allele distributions were compared to those provided in 1000 Genomes European populations [22]. All the genes frequencies examined showed good agreement with Hardy–Weinberg equilibrium.

### 4.4. Statistical Analysis

Quantitative variables are given as medians with interquartile range (IQR) or as a mean ± standard deviation (SD), according to their distribution (Shapiro–Wilk test for normality). For qualitative variables, absolute and relative frequencies are given in percentages.

Associations between polymorphisms and baseline clinical characteristics (Table 1) and tumour response were examined using contingency tables and the Fisher’s exact test. Hazard ratio (HR) and 95% confidence interval (95% CI) were estimated using a Cox proportional hazards model. OS and PFS hazard ratios (HR) and their 95% confidence intervals were estimated for each class and polymorphism. Kaplan–Meier survival curves and long-rank tests were performed to test differences in OS and PFS between patients carrying at least one variant allele (VV and VW) and patients carrying two wild-type alleles as well as for patients carrying two variant alleles (VV) and those carrying none or a single variant allele (VW or WW) for each polymorphism. Kaplan–Meier graphs were generated using the Stata statistical package (STATA IC, software version 14, StataCorp LLC, Texas USA), which was also used for all statistical tests. Significance was set at *p* ≤ 0.05.

## Figures and Tables

**Table 1:** Patient characteristics (n = 57).

Characteristic	Number (%)
Response to bevacizumab	
R (responder)	30 (52.6%)
nR (non responder)	27 (47.4%)
Sex	
Female	16 (28%)
Male	41 (72%)
Age (years)	
<55	13 (22.8%)
55–65	20 (35.1%)
> 65	24 (42.1%)
Metastases,	
Liver, lung, peritoneum or other	
1	16 (27.7%)
2	22 (38.6%)
>2	19 (33.3)
Primary tumour site	
Right	17 (30%)
Left	40 (70%)
Tumour stage at diagnosis,	
I	0
II	2 (3.5%)
III	16 (28.1%)
IV	39 (68.4%)
KRAS status,	
Mutated	42 (76%)
Normal	13 (24%)
Chemotherapy backbone,	
FOLFOX	27 (47%)
FOLFORI	21 (37%)
OTHER (Xeloda, 5-Fu, Irotecan)	9 (16%)
BVZ-regimen	
1	33 (58.9%)
2	15 (26.7%)
3	6 (10.7%)
4	1 (1.8%)
5	1 (1.8%)
Surgical resection	
Yes	20 (35%)
No	37 (65%)

**Table 2:** Genotype frequencies of the SNPs examined and classification of genes according to their biological process (gene ontology). Indicated are allele frequencies of the SNPs analysed found in the 1000 Genomes database [22]. (W = wild-type allele, V = Variant allele).

Gene Ontology	Genetic Variants Type	Genotypes WW WV VV	Treated Patients, n (%)	Alleles (%) W V	1000 Genomes Allele Frequency European
Adenosine pathway	*A2BR* rs2015353 (ADORA2B)	TT	8 (15.09)	T = 0.39 C = 0.60	T = 0.46 C = 0.53
	T > C	TC	26 (49.05)		
	Coding variant	CC	19 (35.85)		
	*ENTPD1* (CD39) rs11188513	CC	6 (10.53)	C = 0.31 T = 0.68	C = 0.36 T = 0.63
	C > T	CT	24 (42.10)		
	Intron variant	TT	27 (47.37)		
	*NT5E* (CD73) rs2229523	AA	3 (5.36)	A = 0.29 G = 0.71	A = 0.29 G = 0.70
	A > G	AG	26 (46.43)		
	Missense variant	GG	27 (48.21)		
Direct angiogenesis	*ANGPT1* rs2445365	GG	33 (57.89)	G = 0.75 A = 0.25	G = 0.75 A = 0.25
	G > A, C	GA	20 (35.09)		
	Intron variant	AA	4 (7.02)		
	*ANGPT2* rs10102851	AA	53 (92.98)	A = 0.97 G = 0.03	A = 0.97 G = 0.03
	A > G	AG	4 (7.02)		
	Intron variant	GG	0		
	*ANGPT2* rs1375668	GG	6 (10.71)	G = 0.27 A = 0.73	G = 0.34 A = 0.66
	G > A, C	GA	18 (32.14)		
	Intron variant	AA	32 (57.14)		
	*ANGPT2* rs2515462	AA	4 (7.02)	A = 0.29 G = 0.71	A = 0.32 G = 0.68
	A > C, G, T	AG	25 (43.86)		
	Intron variant	GG	28 (49.12)		
	*VEGFA* rs833061	CC	10 (17.54)	C = 0.42 T = 0.58	C = 0.50 T = 0.50
	C > G, T	CT	28 (49.12)		
	Upstream transcript variant	TT	19 (33.33)		
	*VEGFA* rs833068	GG	17 (29.82)	G = 0.54 A = 0.46	G = 0.69 A = 0.31
	G > A	GA	28 (49.12)		
	Intron variant	AA	12 (21.05)		
	*VEGFA* rs833069	TT	16 (29.09)	T = 0.54 C = 0.46	T = 0.69 C = 0.31
	T > C, G	TC	27 (49.09)		
	Intron variant	CC	12 (21.82)		
	*VEGFA* rs3025039	CC	43 (75.44)	C = 0.88 T = 0.12	C = 0.88 T = 0.12
	C > T	CT	13 (22.81)		
	3 prime UTR variant	TT	1 (1.75)		
	*FGF2* rs1960669	CC	46 (82.14)	C = 0.90 A = 0.1	C = 0.84 A = 0.16
	C > A	CA	9 (16.07)		
	Intron variant	AA	1 (1.79)		
	*MMP9* rs2236416	AA	43 (75.44)	A = 0.86 G = 0.14	A = 0.83 G = 0.17
	A > G	AG	12 (21.05)		
	Intron variant	GG	2 (3.51)		
	*MMP9* rs2274755	GG	43 (76.78)	G = 0.87 T = 0.13	G = 0.83 T = 0.17
	G > T	GT	12 (21.43)		
	Intron variant	TT	1 (1.79)		
Cytokine signalling	*EDN1* rs5370	GG	34 (60.71)	G = 0.79 T = 0.21	G = 0.78 T = 0.22
	G > T	GT	20 (35.71)		
	Missense variant	TT	2 (3.57)		
	*CCL5* rs2280789	AA	47 (82.46)	A = 0.89 G = 0.11	A = 0.89 G = 0.11
	A > G, C, T	AG	7 (12.28)		
	Intron variant	GG	3 (5.26)		
DNA Topological change	*TOP1* rs34282819	CC	44 (77.19)	C = 0.89 A = 0.11	C = 0.92 A = 0.08
	C > A	CA	13 (22.81)		
	5 prime transcript variant	AA	0		
	*TOP1* rs6072249	AA	18 (31.58)	A = 0.57 G = 0.43	A = 0.55 G = 0.45
	A > G	AG	29 (50.88)		
	Upstream transcript variant	GG	10 (17.54)		
Intracellular signal transduction	*MKNK1* rs8602	CC	29 (53.70)	C = 0.74 A = 0.26	C = 0.72 A = 0.18
	C > A	CA	22 (40.74)		
	Non coding transcript variant	AA	3 (5.56)		
Growth factor	*IGF1* rs6220	GG	4 (7.14)	G = 0.23 A = 0.77	G = 0.27 A = 0.73
	G > A	GA	18 (32.14)		
	3 prime UTR variant	AA	34 (60.71)		

**Table 3:** Genetic heterogeneity of mCRC tumours investigated in this study according to tumour stage. Patient and number of mutated genes according to tumour stage are depicted.

	Number of Mutated Genes (of 13) Analyzed in This Study					
Tumour Stage	<7 genes	7 genes	8 genes	9 genes	10 genes	11 genes
Number of patients with mutated genes (% of total patients in each stage)						
II		1 (50)	1 (50)			
III		4 (25)	5 (31)	5 (31)	2 (13)	
IV	6 (15,5)	11 (28)	6 (15,5)	11 (28)	3 (8)	2 (5)

**Table 4:** Analysis of polymorphisms according to progression-free survival (PFS) and overall survival (OS).

Genetic Variant	PFS		OS	
	Median (Months) (95% CI)	Hazard Ratio *p*-Value	Median (Months) (95% CI)	Hazard Ratio *p*-Value
*A2BR* rs2015353				
TT	6.20 (2.89–16.56)	0.57 (0.26–1.24)	12.52 (4.89-...)	0.70 (0.31–1.60)
TC/CC	10.39 (7.05–15.64)	0.149	28.39 (21.48–34.89)	0.397
*ENTPD1(CD39)* rs11188513				
CC	9.41 (2.43–…)	1.71 (0.65–4.46)	43.84 (12.52–…)	2.32 (0.71–7.54)
CT/TT	8.75 (7.05–13.18)	0.272	23.51 (19.38–30.92)	0.150
*NT5E (CD73)* rs229523				
AA	9.48 (6.62–…)	0.8 (0.25–5.59)	27.54 (11.84-..)	0.59 (0.18–1.95)
AG/GG	9.41 (7.05–13.18)	0.708	23.87 (20.72–34.89)	0.386
*ANGPT1* rs2445365				
GG	9.48 (6.62–14.03)	1.2 (0.689–2.06)	30.92 (21.48–37.87)	1.91 (1.04–3.5)
GA/AA	8.75 (5.74–15.7)	0.519	22.07 (12.52–29.28)	0.034
*ANGPT2* rs10102851				
AA	8.75 (6,89–13.05)	0.97 (0.30–3.15)	-	-
AG/GG	14.03 (12.43–…)	0.953	-	-
*ANGPT2* rs1375668				
GG	10.43 (5.44–…)	0.66 (0.27–1.58)	21.48 (12.20–…)	0.72 (0.24–2.08)
GA/AA	9.41 (7.21–14.03)	0.343	23.87 (20–34.89)	0.544
*ANGPT2* rs2515462				
AA	10.39 (5.74–…)	0.74 (0.26–2.09)	21.48 (12.20–…)	0.82 (0.19–3.51)
AG/GG	8.75 (7.05–13.18)	0.568	27.54 (20–32.89)	0.787
*VEGFA* rs833061				
CC	5.61 (2.79–15.64)	0.71 (0.36–1.43)	21.48 (4.89–32.89)	0.77 (0.37–1.61)
CT/TT	9.48 (7.21–13.18)	0.337	23.87 (18.85–35.02)	0.481
*VEGFA* rs833068				
GG	9.48 (5.44–15.64)	1 (0.56–1.80)	22.2 (14.75–36.52)	0.93 (0.48–1.78)
GA/AA	9.41 (6.62–13.18)	0.999	23.87 (18.85–34.89)	0.821
*VEGFA* rs833069				
TT	8.52 (5.44–15.48)	0.98 (0.54–1.79)	28.39 (14.75–36.52)	1.03 (0.53–2.02)
TC/CC	9.41 (6.62–13.18)	0.946	23.87 (18.62–34.89)	0.926
*VEGFA* rs3025039				
CC	9.41 (6.89–13.18)	1.10 (0.59–2.03)	23.87 (19.38–32.89)	0.84 (0.4–1.75)
CT/TT	8.52 (5.74–16.56)	0.769	27.54(11.74–…)	0.634
*FGF2* rs1960669				
CC	10.95 (7.84–15.70)	3.30 (1.52–7.14)	27.54 (20–34.89)	1.45 (0.69–3.05)
CA/AA	5.44 (2.43–9.48)	**0.001**	22.20 (9.97–32.89)	0.324
*MMP9* rs2236416				
AA	9.48 (7.84–15.64)	2.04 (1.09–3.80)	23.51 (19.38–29.74)	0.91 (0.46–1.81)
AG/GG	6 (2.79–10.95)	**0.022**	32.74 (12.20–43.84)	0.786
*MMP9* rs2274755				
GG	9.48 (7.84–15.64)	1.91 (1.01–3.63)	23.51 (19.38–29.74)	0.9 (0.44–1.84)
GT/TT	6.62 (2.89–10.95)	**0.043**	35.02 (12.20–43.84)	0.780
*EDN1* rs5370				
GG	10.43 (7.21–16.26)	1.29 (0.74–2.26)	23.51 (18.85–34.89)	1.02 (0.55–1.88)
GT/TT	7.84 (4.20–14.03)	0.367	27.54 (11.84–35.02)	0.957
*CCL5* rs2280789				
AA	9.48 (7.21–15.48)	1.44 (0.7–2.98)	27.54 (21.48–32.89)	1.17 (0.49–2.79)
AG/GG	7.44 (2.89–12.43)	0.323	16.03 (7.31-...)	0.723
*TOP1* rs34282819				
CC	8.52 (6.89–12.43)	0.8 (0.42–1.54)	23.87 (20.72–32.72)	0.93 (0.46–1.91)
CA/AA	16.26 (4.20–21.08)	0.501	27.54 (9.97–44.49)	0.853
*TOP1* rs6072249				
AA	11.34 (5.44–18.10)	1.05 (0.58–1.91)	23.51 (12.10–35.02)	0.87 (0.45–1.65)
AG/GG	8.75 (7.05–13.05)	0.862	23.87 (19.38–34.89)	0.662
*MKNK1* rs8602				
CC	8.75 (6.89–13.18)	1.04 (0.59–1.83)	23.51 (16.03–36.52)	1.18 (0.63–2.20)
CA/AA	11.34 (7.21–16.56)	0.904	28.39 (21.48–34.89)	0.614
*IGF1* rs6220				
GG	13.05 (12.43–…)	1.09 (0.38–3.07)	21.48 (4.89–32.89)	1.7 (0.41–7.07)
GA/AA	8.52 (6.62–11.34)	0.874	23.87 (18.85–35.02)	0.457

## Supplementary Materials

The following are available online at https://www.mdpi.com/1422-0067/22/3/1381/s1

## Abbreviations

A2AR	Adenosine A2a receptor
A2BR	Adenosine A2b receptor
ANGPT1	Angiopoietin 1
ANGPT2	Angiopoietin 2
ANGP-TIE	Angiopoietin receptor TEK
BVZ	Bevacizumab
CCL5	C-C motif chemokine ligand 5
CD39	Ectonucleoside triphosphate diphosphohydrolase 1 (ENTPD1)
CRC	Colorectal cancer
CT	Chemotherapy
EDN1	Endothelin 1
FGF2	Fibroblast growth factor 2
IGF1	Insulin like growth factor 1
mCRC	Metastatic colorectal cancer
MKNK1	MAPK interacting serine/threonine kinase 1
MMP9	Matrix metallopeptidase 9
NT5E	5′-Nucleotidase ecto
OS	Overall survival
pCR	Pathological complete response
PFS	Progression-free survival
RECIST	Response Evaluation Criteria in Solid Tumors
RR	Response rate
R	Responders
nR	Non-responders
SNP	Single nucleotide polymorphism
TOP1	Topoisomerase
Tregs	Regulatory T cells
VEGFA	Vascular endothelial growth factor A
